# Gut Bacteria Shared by Children and Their Mothers Associate with Developmental Level and Social Deficits in Autism Spectrum Disorder

**DOI:** 10.1128/mSphere.01044-20

**Published:** 2020-12-02

**Authors:** Yu Chen, Hui Fang, Chunyan Li, Guojun Wu, Ting Xu, Xin Yang, Liping Zhao, Xiaoyan Ke, Chenhong Zhang

**Affiliations:** a State Key Laboratory of Microbial Metabolism, Joint International Research Laboratory of Metabolic and Developmental Sciences, School of Life Sciences and Biotechnology, Shanghai Jiao Tong University, Shanghai, China; b Nanjing Brain Hospital affiliated to Nanjing Medical University, Nanjing, Jiangsu Province, China; c Department of Biochemistry and Microbiology, New Jersey Institute for Food, Nutrition and Health, School of Environmental and Biological Sciences, Rutgers University, New Brunswick, New Jersey, USA; University of Michigan—Ann Arbor

**Keywords:** autism spectrum disorder, gut microbiota, mother-child pair, developmental level, social deficits

## Abstract

The gut microbiota of autism spectrum disorder (ASD) children differs from that of children without ASD. The maternal gut microbiota impacts offspring gut microbiota. However, the relationship between the development of ASD and gut bacteria shared between children and their mothers remains elusive. Our study recruited 76 children with ASD and 47 age- and gender-matched children with typical development (TD), as well as the mothers of both groups, and investigated their gut microbiota using amplicon sequence variants (ASVs). The gut microbiota of ASD children was altered compared with that of children with TD, while no significant alterations were found in their mothers. We established 30 gut bacterial coabundance groups (CAGs) and found the relative abundances of CAG15 and CAG16 significantly decreased in ASD children. CAG15 showed a positive correlation with developmental level. The proportion of ASD children who shared either one of the two *Lachnospiraceae* ASVs from CAG15 with their mothers was significantly lower than that of children with TD. Moreover, we found that CAG12, CAG13, and CAG18 negatively correlated with the severity of social deficits in ASD children. ASD children who shared any one of the four (two *Ruminococcaceae*, one *Lachnospiraceae*, and one *Collinsella*) ASVs in CAG13 and CAG18 with their mothers showed a lower level of social deficits than ASD children that did not share those with their mothers. These data demonstrate that these shared gut bacteria in ASD children are associated with their developmental level and social deficits. This work provides a new direction toward understanding the role of the gut microbiota in the pathogenesis and development of ASD. (This study has been registered in the Chinese Clinical Trial Registry under number ChiCTR-RPC-16008139.)

**IMPORTANCE** Gut microbiota may contribute to the pathogenesis and development of autism spectrum disorder. The maternal gut microbiota influences offspring gut microbial structure and composition. However, the relationship between the clinical symptoms of autism spectrum disorder and the gut bacteria shared between children and their mothers is not yet known. In our study, the gut microbiota of children with autism spectrum disorder differed from that of children with typical development, but there were no differences in the gut microbiota of their mothers. More importantly, gut bacteria shared between children with autism spectrum disorder and their mothers were related to developmental disabilities and social deficits. Thus, our study suggests that these shared gut bacteria may play an important role in the development of autism spectrum disorder. This provides a new direction for future studies aiming to explore the role of the gut microbiota in autism spectrum disorder.

## Abstract (precis)

Gut microbiota may contribute to the pathogenesis and development of autism spectrum disorder. The maternal gut microbiota influences offspring gut microbial structure and composition.

## INTRODUCTION

Autism spectrum disorder (ASD), first described by Leo Kanner in 1943, is a set of neurodevelopmental disorders (1). The core symptoms of ASD are social deficits, restricted interests, and repetitive behaviors (2). Developmental disabilities are common complications in children with ASD (3). According to epidemiological data from the United States, Western Europe, and Asia Pacific, the prevalence of ASD is 1.5% to 2.2% in children aged 3 to 17 years (4–6). The exact biological mechanisms of ASD are still unclear, but genetic, epigenetic, and environmental factors are all reported to have possible roles in the pathogenesis of ASD (7).

Emerging evidence suggests that the gut microbiota may be an important factor in the development of ASD. The structure and composition of gut microbiota in ASD children differ from those in healthy controls (8–10). Microbiota transfer therapy (MTT) and dietary modulation of gut microbiota can both alleviate gastrointestinal (GI) dysfunction and behavioral symptoms in children with ASD (11, 12). Gut microbiota can modulate the development of the central nervous system (CNS) through the brain-gut-microbiota (BGM) axis, and this may influence the development of ASD (13). The strain Bacteroides fragilis NCTC 9343 was reported to reduce the level of chronic inflammation in the offspring of mice with immune activation, which alleviated ASD-like behavioral abnormalities such as defects in communication and stereotypic behaviors (14). Buffington et al. showed that the strain Lactobacillus reuteri MM4-1A (ATCC PTA-6475) ameliorated the ASD-like behavioral abnormalities in offspring of obese mice via oxytocin stimulation (15). Moreover, 5-aminovaleric acid (5AV) and taurine, two gut microbial metabolites, alleviated social deficits and repetitive behaviors in the offspring of mice transplanted with the gut microbiota from ASD patients (16). Collectively, these findings suggest that alterations in the gut microbiota may contribute to the pathophysiology of ASD.

A clinical study showed that the gut microbial alterations in ASD children were consistent with those in their mothers (17), suggesting that alterations in maternal gut microbiota are related to the increased risk of ASD in children. A possible mechanism to explain this finding is that maternal microbes impact the heath of offspring by vertical transmission. In mammals, maternal microbes can be transmitted to the offspring via natural childbirth, dermal contact, breast feeding, etc. (18). This transmission is critical in establishing the gut microbial structure and composition in the offspring (19) and therefore impacts gut microbial functions in the offspring, which include processes affecting growth (20), regulation of the immune system (21), and cerebral development (22). Conversely, alterations in this maternal transmission are associated with the development of human diseases, including neurodevelopmental disorders (23). Thus, the relationship between the gut bacteria shared by children and their mothers and the development of ASD merits further investigation.

To answer the questions above, 76 children with ASD and their mothers (MA) were recruited to the study along with 47 age- and gender-matched children with typical development (TD) and their mothers (MT). The gut microbiota of these groups were analyzed by sequencing the V3-V4 regions of the bacterial 16S rRNA gene in fecal samples. Gut microbiota of children with ASD differed from that of children with TD, but no significant differences were found between the gut microbiota of their mothers. Gut bacteria shared between children with ASD and their mothers were found to be associated with ASD behavioral symptoms.

## RESULTS

### Developmental level and gastrointestinal symptoms in ASD children.

In the Children’s Mental Health Research Center of the Affiliated Brain Hospital of Nanjing Medical University, 76 children (2.6 to 8.2 years old) were screened using the childhood autism rating scale (CARS) and were diagnosed with ASD based on the autism diagnostic interview (ADI-R) and autism diagnostic observation schedule (ADOS) (see Table S1 in the supplemental material). Forty-seven children with TD (2.5 to 5.9 years old) in Nanjing were also recruited to the study. The age and sexual ratios were not significantly different between the ASD and TD groups (Table 1), and there were no significant differences in mode of delivery in childbirth, body height, body weight, body mass index (BMI), head circumference, and waist/hip ratio between the two groups. In addition, no significant differences in intake of total energy, protein, fat, carbohydrate, fiber, vitamin A, vitamin B_1_, vitamin C, Na, Ca, Mg, Fe, and Zn were found between the ASD and TD groups (see Table S2). However, waistline and hipline measurements of the ASD group were significantly lower than those of the TD group (Table 1). The ASD group showed a significantly lower level of vitamin B_2_ intake than the TD group (Table S2). The GI severity index in the ASD group was significantly higher than that in the TD group (see Fig. S1). The proportion of ASD children (91.4%) with GI symptoms was significantly higher than that of children with TD (69%). The proportions of ASD children with constipation and unexplained daytime irritability were significantly higher than those of children with TD (50.0% versus 23.8% for constipation and 48.6% versus 16.7% for unexplained daytime irritability) (see Table S3). Development quotient (DQ) scores of adaptive behavior, gross motor, fine motor, language, and personal social behavior, the five parameters of the Gesell developmental scale (GDS), were significantly decreased in the ASD group compared to scores in the TD group (Table 2), suggesting that the developmental level of ASD children was lower than that of children with TD.

**TABLE 1:** General anthropometric parameters in the ASD and TD groups

Parameter	Values for children with:^*a*^		*P* value^*b*^
	ASD (*n *= 76)	TD (*n *= 47)	
Age (yrs) (*n*, mean ± SEM)^*c*^	76, 3.96 ± 0.12	47, 4.25 ± 0.12	0.15
Gender (*n*, %)^*c*^			0.50
Male	61, 80.26	41, 87.23	
Female	15, 19.74	6, 12.77	
Mode of delivery (*n*, %)			0.18
Eutocia	32, 47.46	25, 60.98	
Cesarean	35, 52.24	16, 39.02	
Measurements (*n*, mean ± SEM)			
Height (cm)	70, 105.31 ± 1.02	46, 106.67 ± 1.215	0.39
Weight (kg)	69, 17.70 ± 0.57	46, 18.89 ± 0.71	0.18
BMI^*d*^	67, 15.78 ± 0.34	46, 16.37 ± 0.32	0.23
Head circumference (cm)	71, 50.52 ± 0.21	45, 50.66 ± 0.20	0.65
Waistline (cm)	67, 49.82 ± 0.69	45, 53.06 ± 1.00	0.002
Hipline (cm)	67, 54.41 ± 0.62	45, 58.08 ± 1.03	0.003
Waist/hip ratio	66, 0.92 ± 0.01	45, 0.91 ± 0.01	0.56

a TD, typical development; ASD, autism spectrum disorder.

b Pearson chi-square was used to analyze variations in gender and mode of delivery between the ASD and TD groups, and Student’s *t* test was utilized to analyze changes in other clinical outcomes.

c The data were measured as in Li et al.’s study (47).

d BMI, body mass index.

**TABLE 2:** Developmental levels

Parameter of GDS^*a*^	Developmental quotient score (mean ± SEM)^*b*^	
	ASD group (*n *= 76)	TD group (*n *= 46)
Adaptive behavior^*c*^	66.05 ± 1.86	101.37 ± 1.86
Gross motor^*c*^	74.07 ± 1.58	102.26 ± 1.96
Fine motor^*c*^	71.61 ± 1.85	99.80 ± 1.75
Language^*c*^	54.16 ± 2.52	108.00 ± 2.49
Personal social behavior^*c*^	57.26 ± 1.87	110.83 ± 3.70

a GDS (Gesell developmental scale) measured according to Li et al.’s study (47).

b TD, typical development; ASD, autism spectrum disorder.

c *P* < 0.01 by Student’s *t* test.

10.1128/mSphere.01044-20.1 TABLE S1 Scores of CARS, ADI-R, and ADOS in the ASD group. Data are presented as means ± standard errors of the means (SEMs). CARS, childhood autism rating scale, *n* = 76; ADI-R, autism diagnostic interview, *n* = 76; communication, *n* = 76; VC, verbal communication, *n* = 47; NVC, nonverbal communication, *n* = 29; RSI, reciprocal social interaction, *n* = 76; RBSP, repetitive behavior and stereotyped patterns, *n* = 76; ADOS, autism diagnostic observation schedule, *n* = 76; ASD, autism spectrum disorder. The data for CARS, ADI-R, and ADOS were measured as in Li et al.’s study (47). Download Table S1, DOCX file, 0.02 MB. Copyright © 2020 Chen et al. 2020 Chen et al. This content is distributed under the terms of the Creative Commons Attribution 4.0 International license.

10.1128/mSphere.01044-20.2 TABLE S2 Dietary intake of the ASD and TD groups for 24 h before the collection of fecal samples. Data are shown as means ± SEMs. Student’s *t* tests were performed to analyze the variations between the ASD and TD groups. **, *P* < 0.01; TD, typical development; ASD, autism spectrum disorder. Download Table S2, DOCX file, 0.02 MB. Copyright © 2020 Chen et al. 2020 Chen et al. This content is distributed under the terms of the Creative Commons Attribution 4.0 International license.

10.1128/mSphere.01044-20.3 TABLE S3 GI symptoms in the ASD and TD groups. Data are presented as *n* of children in the ASD and TD groups. Pearson chi-square tests were used to analyze differences. **, *P* < 0.01; ***, *P* < 0.001; ASD, autism spectrum disorder; TD, typical development. Download Table S3, DOCX file, 0.02 MB. Copyright © 2020 Chen et al. 2020 Chen et al. This content is distributed under the terms of the Creative Commons Attribution 4.0 International license.

10.1128/mSphere.01044-20.5 FIG S1 A higher level of GI severity in ASD children. The boxes represent the interquartile ranges, lines inside the boxes stand for medians, and whiskers represent the minimum and maximum values. ***, *P* < 0.001 by Student’s *t* test; TD, typical development, *n *= 42; ASD, autism spectrum disorder, *n *= 70. Download FIG S1, DOCX file, 0.2 MB. Copyright © 2020 Chen et al. 2020 Chen et al. This content is distributed under the terms of the Creative Commons Attribution 4.0 International license.

### Gut microbiota in ASD children and their mothers.

**FIG 1:**
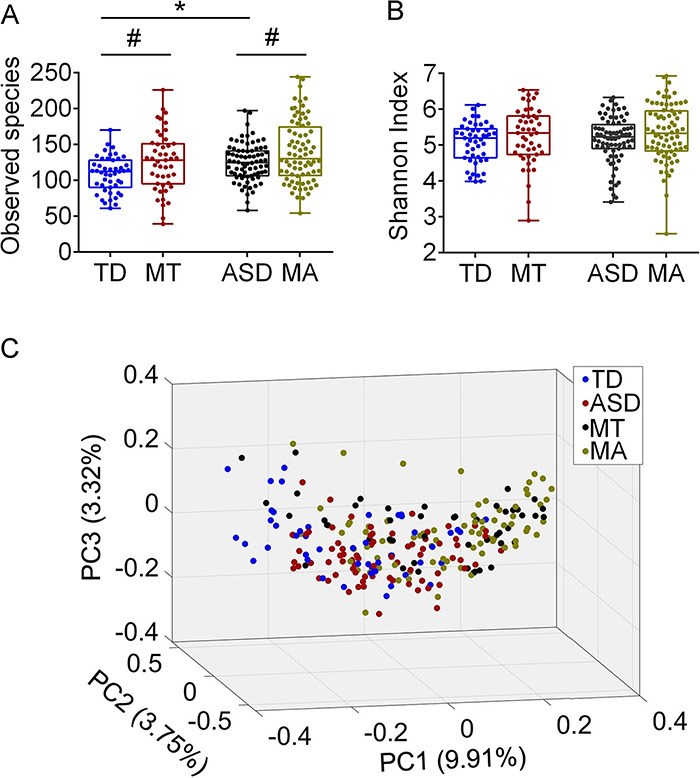
The gut microbial diversity, richness, and structures in ASD children and their mothers and in children with TD and their mothers. Observed species (A) and Shannon indexes (B) among the ASD, TD, MA, and MT groups. The boxes represent the interquartile ranges, lines inside the boxes stand for medians, and whiskers represent the minimum and maximum values. Student’s *t* tests were used to analyze the variations in ASD versus TD and MA versus MT, while paired *t* tests were used in ASD versus MA and TD versus MT. *, *P* < 0.05 for comparison in ASD versus TD and MA versus MT; *#*, *P* < 0.05 for comparison in ASD versus MA and TD versus MT. (C) Principal-coordinate analysis plot of the gut microbiota in the TD, ASD, MT, and MA groups based on Bray-Curtis distance. Data in panel C were processed by log(10) transformation. TD, children with typical development (*n *= 47); ASD, autism spectrum disorder children (*n *= 76); MT, mothers of children with TD (*n *= 47). MA, mothers of ASD children (*n *= 76).

Gut microbial alterations in ASD children and their mothers (MA) (28 to 41 years old) were explored in comparison with those in children with TD and their mothers (MT) (30 to 40 years old) by sequencing the bacterial 16S rRNA gene V3-V4 regions in fecal samples from these groups. Totally, 5,755,973 high-quality sequencing reads (23,398 ± 4,753 reads per sample) were denoised into 3,642 amplicon sequence variants (ASVs). Richness (observed species) of the gut microbiota in the ASD group was significantly greater than that in the TD group (Fig. 1A). There was no significant difference in richness between the MA and MT groups. Both the ASD and TD groups had significantly lower gut microbial richness than their mothers (Fig. 1A). No significant differences in gut microbial diversity (Shannon index) were observed among the ASD, MA, TD, and MT groups (Fig. 1B).

Principal-coordinate analysis (PCoA) of the Bray-Curtis distance based on the ASV data revealed that the overall structure of the gut microbiota in the ASD group significantly differed from that in the TD group (Fig. 1C and see Fig. S2A) (*P* = 0.002 with permutational multivariate analysis of variance [PERMANOVA], 9,999 permutations), but there was no significant difference in the gut microbial structures between the MA and MT groups (Fig. S2B). Compared with those of their mothers, the ASD and TD groups had significantly different gut microbial structures (*P* = 0.001 versus *P* = 0.001, respectively, with PERMANOVA, 9,999 permutations). Based on Bray-Curtis distance, we showed that the similarity of gut microbial structures between ASD children and their mothers was higher than that between children with TD and their mothers (see Fig. S3).

10.1128/mSphere.01044-20.6 FIG S2 Principal-coordinate analysis plots of the gut microbiota in TD versus ASD (A) and MT versus MA (B) based on Bray-Curtis distance. Data were processed by log(10) transformation. TD, children with typical development, *n *= 47; ASD, children with autism spectrum disorder, *n *= 76; MT, mothers of children with TD; *n *= 47; MA, mothers of ASD children, *n *= 76. Download FIG S2, DOCX file, 0.8 MB. Copyright © 2020 Chen et al. 2020 Chen et al. This content is distributed under the terms of the Creative Commons Attribution 4.0 International license.

10.1128/mSphere.01044-20.7 FIG S3 Higher similarity of gut microbial structures between ASD children and their mothers than between TD children and their mothers. Bray-Curtis distance was used to calculate the distance between values for children and for their mothers. The boxes represent the interquartile ranges, lines inside the boxes stand for medians, and whiskers represent the minimum and maximum values. **, *P* < 0.01 by Student’s *t* test; TD, typical development, *n *= 47. ASD, autism spectrum disorder, *n *= 76. Download FIG S3, DOCX file, 0.3 MB. Copyright © 2020 Chen et al. 2020 Chen et al. This content is distributed under the terms of the Creative Commons Attribution 4.0 International license.

**FIG 2:**
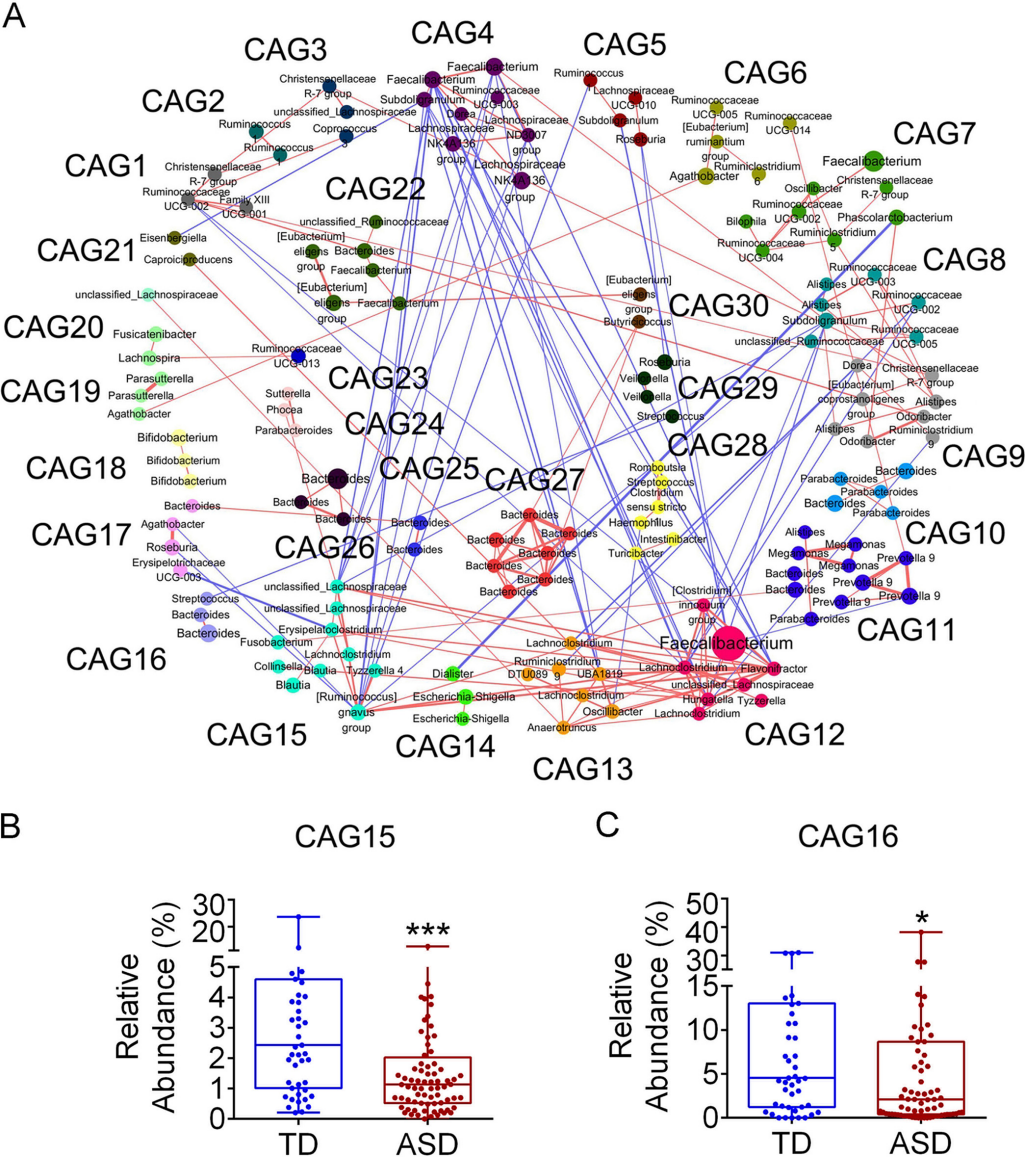
Gut bacterial coabundance groups (CAGs) of 261 ASVs shared by >10% of children in the ASD and TD groups. (A) Amplicon sequence variant (ASV)-level network diagram of the 261 ASVs. Node size stands for the mean abundance of each ASV, with line width indicating correlation magnitude. Red lines or blue lines between nodes represent positive or negative correlations between the nodes they connect, respectively. Only lines corresponding to correlations with a magnitude greater than 0.35 are drawn. The 261 ASVs are clustered into 30 gut bacterial CAGs using the WGCNA package in R. Compared with those in the TD group, the relative abundances of CAG15 (B) and CAG16 (C) in the ASD group were significantly decreased. Boxes and whiskers are denoted as for Fig. 1. Mann-Whitney test was used to analyze the variations. *, *P* < 0.05, ***, *P* < 0.001. TD, children with typical development (*n *= 47); ASD, autism spectrum disorder children (*n *= 76).

Alterations in gut microbial compositions of children with ASD were further explored via the construction of a coabundance network based on Spearman’s correlation coefficients among the 261 ASVs that existed in >10% of children in the ASD and TD groups (Fig. 2A). These ASVs were clustered into 30 coabundance groups (CAGs) (see Table S4). Relative abundances of CAG15 and CAG16 were significantly lower in the ASD group than in the TD group (Fig. 2B and C; see also Fig. S4). CAG15 mainly contained ASVs belonging to the family *Lachnospiraceae*, while CAG16 predominantly comprised ASVs from the family *Lachnospiraceae* and the genus *Bacteroides* (Table S4).

10.1128/mSphere.01044-20.4 TABLE S4 Taxonomical assignments of amplicon sequence variants (ASVs) in 30 coabundance groups (CAGs). Download Table S4, PDF file, 0.04 MB. Copyright © 2020 Chen et al. 2020 Chen et al. This content is distributed under the terms of the Creative Commons Attribution 4.0 International license.

10.1128/mSphere.01044-20.8 FIG S4 Relative abundances of CAGs between the ASD and TD groups. The boxes represent the interquartile ranges, lines inside the boxes stand for medians, and whiskers represent the minimum and maximum values. Mann-Whitney test was used to analyze the variations between the two groups. CAG, coabundance group; TD, typical development, *n *= 47; ASD, autism spectrum disorder; *n *= 76. Download FIG S4, DOCX file, 1.2 MB. Copyright © 2020 Chen et al. 2020 Chen et al. This content is distributed under the terms of the Creative Commons Attribution 4.0 International license.

### Association of gut bacteria shared between ASD children and their mothers with developmental level.

**FIG 3:**
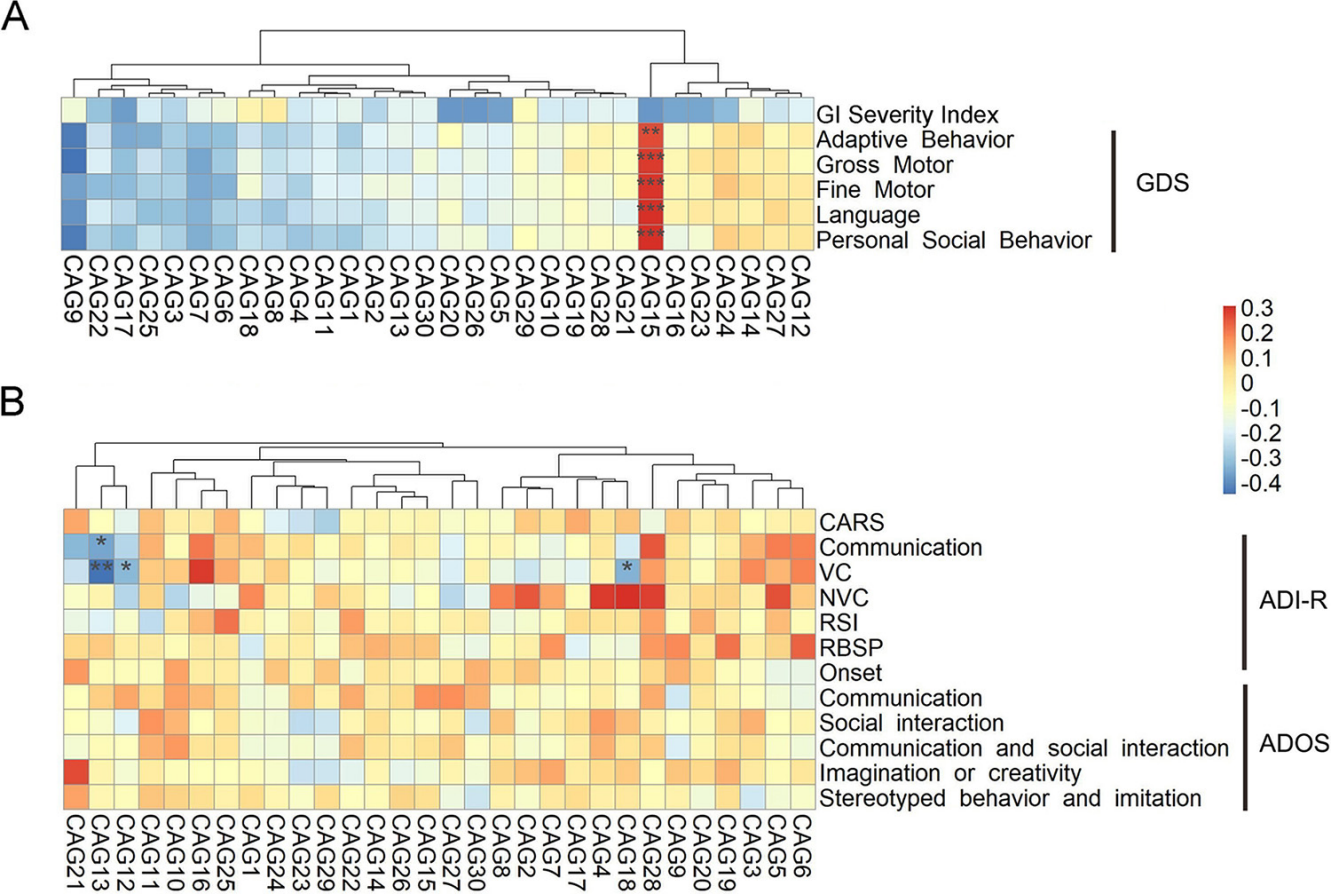
Heat maps of correlations between gut bacterial CAGs and clinical parameters of ASD. (A) GI severity index and GDS in the ASD and TD groups. (B) CARS, ADI-R, and ADOS in the ASD group. The color of the cells represents Spearman’s correlation coefficient between each CAG and clinical parameter. *, *P* < 0.05; **, *P* < 0.01; ***, *P* < 0.001 (adjusted according to Benjamini and Hochberg [53]). GI severity index, *n *= 112; GDS, Gesell developmental scale, *n *= 122; CARS, childhood autism rating scale, *n *= 76; VC, verbal communication, *n *= 47; NVC, nonverbal communication, *n *= 29. Reciprocal social interaction (RSI) and repetitive behavior and stereotyped patterns (RBSP) in ADI-R (autism diagnostic interview-revised) and ADOS (autism diagnostic observation schedule), *n *= 76; CAG, coabundance group; TD, children with typical development; ASD, autism spectrum disorder children.

The relationship of gut microbiota to GI severity and developmental level, which were significantly altered in the ASD group, was examined by calculating Spearman’s correlation coefficients in gut bacterial CAGs versus the GI severity index and CAGs versus GDS in the ASD and TD groups. None of the CAGs showed a significant correlation with the GI severity index, while the relative abundance of CAG15 showed a significant positive correlation with DQ scores of adaptive behavior, gross motor, fine motor, language, and personal social behavior, the five parameters of the GDS (Fig. 3A).

In our work, if an ASV was detected in a child and his/her mother, then the ASV was considered shared in this child-mother pair. In other words, if an ASV was detected in either a child or his/her mother, or was not detected in either of them, we considered that the ASV was not shared in this mother-child pair. For CAG15, 10 ASVs were shared in child-mother pairs. The proportion of children with ASD who shared any one of these 10 ASVs with their mothers was then compared with that of children with TD who shared this ASV with their mothers (Table 3). The proportions of ASD children that shared *Lachnospiraceae* ASV3491 and *Lachnospiraceae* ASV790 (Table S4) with their mothers were significantly lower than those of children with TD (13.2% versus 27.7%, respectively, for ASV3491 and 2.6% versus 12.8%, respectively, for ASV790).

**TABLE 3:** Numbers of children with ASD and TD that shared ASVs in CAG15 with their mothers

ASV ID	No. of children with ASVs shared or not with their mother^*a*^			
	ASD group (*n *= 76)		TD group (*n *= 47)	
	Shared	Not shared	Shared	Not shared
ASV3051	70	6	45	2
ASV406	31	45	15	32
ASV3419^*b*^	10	66	13	34
ASV790^*b*^	2	74	6	41
ASV4196	2	74	3	44
ASV3792	2	74	0	47
ASV1765	2	74	2	45
ASV493	2	74	4	43
ASV4568	1	75	1	46
ASV2839	0	76	2	45

a TD, typical development; ASD, autism spectrum disorder.

b *P* < 0.05 by Pearson chi-square test.

### Association of gut bacteria shared between ASD children and their mothers with ASD score symptoms.

The association of members of the gut microbiota with ASD core symptoms, including social deficits, restricted interests, and repetitive behaviors, was explored by calculating correlations between gut bacterial CAGs and scores for CARS, ADI-R, and ADOS in the ASD group. CAG13 showed a significant negative correlation with communication in the ADI-R, while CAG12, CAG13, and CAG18 each showed significant negative correlations with verbal communication (VC) in the ADI-R (Fig. 3B). This suggests that CAG13, CAG12, and CAG18 may be beneficial in alleviating the social deficits of children with ASD. CAG12 and CAG13 contained ASVs from the families *Ruminococcaceae* and *Lachnospiraceae*, while ASVs in CAG18 belonged to the genera *Bifidobacterium* and *Collinsella* (Table S4).

**FIG 4:**
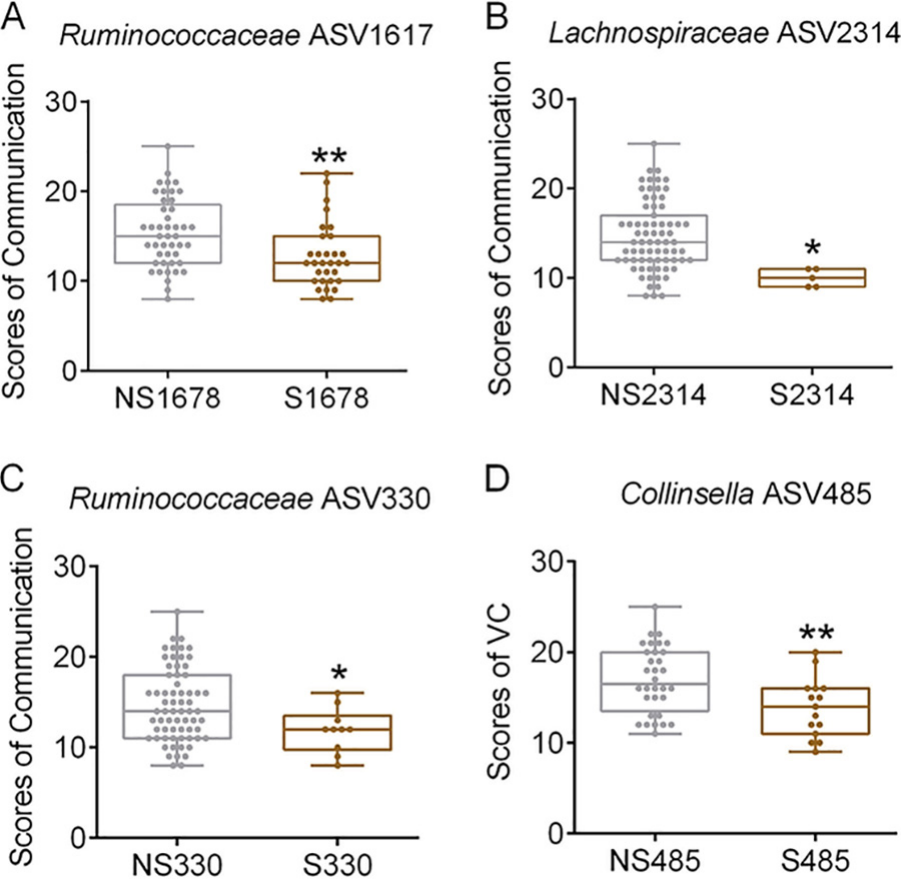
Alterations in children’s gut bacteria shared with their mothers with the severity of social deficits. The ASD group who shared ASV1617 (A), ASV2314 (B), or ASV330 (C) with their mothers showed significantly decreased scores of communication in the autism diagnostic interview (ADI-R). (D) The ASD group who shared ASV485 with their mothers showed significantly decreased scores of VC. Boxes and whiskers are denoted as for Fig. 1. Student’s *t* test was used to analyze the variations. *, *P* < 0.05; **, *P* < 0.01. NS1678, children who do not share ASV1678 with their mothers in the ASD group, *n *= 45; S1678, children who share ASV1678 with their mothers in the ASD group, *n *= 31; NS2314, children who do not share ASV2314 with their mothers in the ASD group, *n *= 71; S2314, children who share ASV2314 with their mothers in the ASD group, *n *= 5; NS330, children who do not share ASV330 with their mothers in the ASD group, *n *= 66; S330, children who share ASV330 with their mothers in the ASD group, *n *= 10; NS485, children who do not share ASV485 with their mothers in the ASD group, *n *= 32; S485, children who share ASV485 with their mothers in the ASD group, *n *= 15; VC, verbal communication; TD, children with typical development; ASD: autism spectrum disorder children.

For CAG12, CAG13, and CAG18, the number of ASVs shared in ASD child-mother pairs were 8, 10, and 7, respectively. ASD children who shared any one of these ASVs in CAG12, CAG13, and CAG18 with their mothers were annotated as “shared” the ASV (S plus the identifier [ID] of the ASV) group, while ASD children who did not share the ASV with their mothers were annotated as “no-shared” the ASV (NS plus ID of the ASV) group. For example, ASD children who shared ASV1617 with their mothers were annotated as the S1617 group, and ASD children who did not share the ASV with their mothers were annotated as the NS1617 group. The social deficits of these S groups and their NS groups were then compared. The S1617, S2314, and S330 groups had significantly decreased scores of communication compared with those for the NS1617, NS2314, and NS330 groups, respectively (Fig. 4A, B, and C). *Ruminococcaceae* ASV1617, *Lachnospiraceae* ASV2314, and *Ruminococcaceae* ASV330 were all located in CAG13 (Table S4). In addition, the S485 group showed significantly lower scores of VC than the NS485 group (Fig. 4D), and *Collinsella* ASV485 was from CAG18.

In summary, we found specific gut bacteria that were shared between ASD children and their mothers and which related to social deficits.

## DISCUSSION

In this study, we found an altered gut microbiota in ASD children compared with that in age- and gender-matched children with TD, while there were no significant differences with the gut microbiota of their mothers. The similarity of gut microbial structure between ASD children and their mothers was higher than that between children with TD and their mothers. Furthermore, specific gut bacteria shared between children and their mothers were associated with neurodevelopmental level and social deficits in children with ASD.

In our work, the composition of gut microbiota in children with ASD was different from that of age- and gender-matched children with TD. For example, the numbers of specific gut bacteria, which were predominantly from the family *Lachnospiraceae* and the genus *Bacteroides*, decreased in ASD children. These results were consistent with previous studies that reported the abundances of this family and genus were decreased in Chinese ASD children (24, 25). However, a previous study, involving a Spanish cohort, showed that the abundance of *Bacteroides* increased in ASD children, which is inconsistent with our finding (26). On the other hand, our study also found the specific ASVs belonging to *Lachnospiraceae* and *Bacteroides* were not significantly different between ASD children and those with TD (see Fig. S4 and Table S4 in the supplemental material). This suggested that the alterations in ASVs which were from the same family or genus in ASD children may be different. These differences might be attributable to species- or even strain-specific functions of the bacteria (27). Future studies should examine the relationship between the gut microbiota and the development of ASD at the strain level.

Previous studies showed that coabundance analysis may be a more ecologically relevant method to identify the critical members of the gut microbiota involved in human disease (28, 29). Using this type of analysis, the specific gut bacteria, which mainly belonged to *Lachnospiraceae*, *Ruminococcaceae*, and *Bifidobacterium*, were found to be negatively correlated with the severity of ASD behavioral symptoms, including developmental delay and social deficits. Deficiencies of these bacteria have been reported to play a role in ASD behavioral abnormalities. Many members of the family *Lachnospiraceae* are known as butyrate-producing bacteria (30, 31). A previous study revealed that butyrate alleviated the acylation deficiency (caused by the mutations of in the bromodomain- and PHD finger-containing protein 1 *Brpf1* gene) of histone H3 at lysine 23 in mouse embryos and fibroblasts and human embryonic kidney 293 cells, and this deficiency was also found in children with neurodevelopmental delay (32). This study suggests that butyrate may alleviate the severity of neurodevelopmental delay in children. Many members of the family *Ruminococcaceae* produce acetate (33–37), which is reported to be decreased in ASD children (24). Acetate was shown to decrease the permeability of the blood-brain barrier (BBB) in germfree mice (38), which alleviated the severity of social deficits in a mouse model of ASD (39). The genus *Bifidobacterium* contains gamma-aminobutyric acid (GABA)-producing bacteria (40, 41). A previous study showed that excessive sequestration of GABA into the mitochondria of cerebral cells caused social deficits in *Drosophila*, while release of the sequestrated GABA or increase in the level of GABA in the brain alleviated these social deficits (42). Thus, the gut bacteria identified as related to ASD behavioral symptoms in this study may contribute to the pathophysiology and development of ASD. In the future, we should explore the mechanisms by which these gut bacteria alleviate the behavioral symptoms of ASD.

A previous study, involving a Chinese cohort, reported that the gut microbiota of ASD children were different from those of healthy children, and mothers of ASD children also had altered gut microbiota compared to those of mothers of healthy children. Moreover, alterations in gut microbiota of ASD children and their mothers were consistent (17). Our study found no significant differences in maternal gut microbiota of children with ASD and children with TD. Deficiencies in specific gut bacteria were shared between children and their mothers and were related to the ASD behavioral symptoms. Together, our findings suggest that vertical transmission of gut microbiota between children with ASD and their mothers may be altered. Previous studies demonstrated that alterations in vertical transmission of maternal gut microbiota, such as those caused by caesarean section, formula feeding, and antibiotic exposure during delivery, led to the deficiencies in potential beneficial bacteria in their children. These microbial deficiencies are associated with increased risks of many human diseases, including neurodevelopmental disorders (43–46). Therefore, long-term follow-up clinical studies should be performed with a larger cohort to validate and understand the mechanisms of interaction between this vertical transmission of gut microbiota and the pathophysiology and development of ASD. We did not obtain maternal GI and dietary data from these children with ASD and TD, which is a limitation of our study.

In conclusion, our work demonstrates that the gut microbiota is altered in ASD children but not in their mothers. In addition, gut bacteria shared by children and their mothers is associated with developmental level and social deficits in children with ASD. Children’s gut bacteria shared with their mothers may play an important role in the development of ASD, providing a new direction for future studies aiming to explore the role of the gut microbiota in ASD.

## MATERIALS AND METHODS

### Clinical investigation.

This trial was registered in the Chinese Clinical Trial Registry under the registration number ChiCTR-RPC-16008139. All participants completed an informed consent form, and the procedures of this study were approved by the Institutional Review Board of Nanjing Brain Hospital of Nanjing Medical University (permit number 2016-KY017). This clinical trial recruited 184 children and mothers of 123 of these children. Participants in our study comprised 123 children and their mothers, while participants in Li et al.’s study were the 184 children (47).

Seventy-six children with ASD and their mothers were recruited to the trial. The children were screened by CARS and diagnosed with ASD by ADI-R and ADOS in the Children’s Mental Health Research Center of the Nanjing Brain Hospital affiliated with Nanjing Medical University. Then, 47 children with TD from Ying Hua kindergarten (Nanjing) and their mothers were recruited. Children with ASD who suffered from other neurological conditions, such as epilepsy and attention deficit hyperactivity disorder (ADHD), and serious organic diseases, such as hepatorenal function disorder, were excluded. Children with TD were confirmed by at least two clinicians as not having any neurodevelopmental disorders, serious organic disease, such as hepatorenal function disorders, or any gastrointestinal surgery.

Clinical parameters, including general anthropometric parameters, GDS, CARS, ADI-R, and ADOS, of children were measured based on the methods described in previous studies (29, 48, 49). GI symptoms in both groups of children were evaluated based on GI severity index, including constipation, diarrhea, average stool consistency, stool smell, flatulence, abdominal pain, unexplained daytime irritability, nighttime awakening, and abdominal tenderness (during examination), as described in a previous study (50). A 24-h dietary record was used to estimate nutritional intake of the ASD and TD groups for 24 h before the collection of fecal samples based on the China Food Composition 2009, as previously described (28).

Tools for fecal collection were provided to participants who had not suffered from fever or used antibiotics or any other medications for more than 3 days in the previous month. Maternal fecal samples were collected the next day, and samples from children were collected within 1 week of the psychological assessments. Fecal samples stored in stool collection tubes with DNA stabilizer (1038111300; Stratec Molecular, Germany) were collected in participants’ homes by their mothers (children) or themselves. The tubes with DNA stabilizer protect gut bacteria in fecal samples at room temperature for at least 24 h. Fecal samples received at the Children’s Mental Health Research Center of the Affiliated Brain Hospital of Nanjing Medical University were immediately stored at −80°C until fecal DNA extraction.

### Fecal DNA extraction and 16S rRNA gene V3-V4 region sequencing.

Gut microbial DNA in fecal samples was extracted using methods reported in previous studies. An Illumina MiSeq platform (Illumina, Inc., USA) with MiSeq reagent kit v3 (600-cycle) (MS-102-3033; Illumina, USA) was used to sequence the 16S rRNA gene V3-V4 regions of gut bacteria in fecal samples. A two-step amplification (amplification of 16S rRNA gene V3-V4 region and the index PCR) was used to prepare the library according to the manufacturer’s instructions (part number 15044223 rev. B; Illumina) with modifications described by Zhang et al. (51).

### Bioinformatics analysis of sequencing data.

Sequence data from the Illumina MiSeq platform were inputted to QIIME2 as type “SampleData[PairedEndSequencesWithQuality],” and all steps of sequence processing and quality control were performed in QIIME2 (version 2018.06). Adapter sequences were trimmed by using the script “qiime cutadapt trim-paired.” The DADA2 pipeline, including error filtering, trimming, denoising, merging of paired reads, and removal of chimeras, was used to cluster high-quality reads into amplicon sequence variants (ASVs) which were annotated using the SILVA reference database (version 132). These reads of each sample were downsized to 10,000 to normalize the even sampling depths.

### Clustering of gut bacterial coabundance groups.

Correlation relationships among 261 ASVs, which existed in >10% of children with ASD and children with TD, were calculated by using the Spearman’s correlation coefficient (*R*) based on their relative abundance. This *R* matrix, also known as topological overlap matrix (TOM), was converted to a correlation distance (1 − *R*) matrix, also known as dissTOM, in the R (version 3.5.1) package WGCNA (52). Using this package, a hierarchical clustering tree of these ASVs based on dissTOM was produced by using the Ward.D2 clustering algorithm. Based on this hierarchical clustering tree, the 261 ASVs were clustered into 30 CAGs. The CAG network was visualized in Cytoscape (version 3.2.1).

### Statistical analysis.

Student’s *t* test and chi-square test, performed using the statistical software SPSS22.0 (IBM Corp., Armonk, NY, USA), were used to analyze the clinical parameters of volunteers. Permutational multivariate analysis of variance (PERMANOVA) was utilized to evaluate the significance of the differences in gut microbial structures in ASD, TD, MA, and MT groups in QIIME2. To adjust the *P* value for multiple testing, false-discovery rate (FDR) values were estimated using the Benjamini-Yekutieli method in correlation analysis between CAGs and clinical parameters with Spearman’s algorithm. *P* values of <0.05 were regarded as statistically significantly different.

### Data availability.

The 16S rRNA V3-V4 amplicon sequencing data are available in the NCBI Short Read Archive (SRA) repository under accession number PRJNA644763.
