# Supplementary material for: Gut Bacteria Shared by Children and Their Mothers Associate with Developmental Level and Social Deficits in Autism Spectrum Disorder
Source: mSphere. 2020 Dec 2;5(6):e01044-20. doi: 10.1128/mSphere.01044-20 (PMC7716279; doi:10.1128/mSphere.01044-20)
Supplement: TABLE S1 [file mSphere.01044-20-st001.docx]

|  | **Scores** | **Score Range** | **Cut-off Score** |
| --- | --- | --- | --- |
| **CARS** | 37.4 ± 0.4 | 32-50 | 30 |
| **ADI-R** |  |  |  |
| Communication | 14.3 ± 0.4 | 8-25 | 7 |
| VC | 16.0 ± 0.6 | 9-25 | 8 |
| NVC | 11.5 ± 0.4 | 8-16 | 7 |
| RSI | 19.6 ± 0.5 | 10-31 | 10 |
| RRB | 6.3 ± 0.3 | 2-14 | 3 |
| Onset | 3.8 ± 0.1 | 1-5 | 1 |
| **ADOS** |  |  |  |
| Communication | 6.5 ± 0.2 | 3-10 | 3 |
| Social interaction | 11.3 ± 0.2 | 6-14 | 4 |
| Communication and social interaction | 17.7 ± 0.3 | 9-22 | 8 |
| Imagination or creativity | 2.7 ± 0.1 | 1-4 | - |
| Stereotyped behavior and imitation | 3.9 ± 0.1 | 2-6 | - |
