# Supplementary material for: Gut Bacteria Shared by Children and Their Mothers Associate with Developmental Level and Social Deficits in Autism Spectrum Disorder
Source: mSphere. 2020 Dec 2;5(6):e01044-20. doi: 10.1128/mSphere.01044-20 (PMC7716279; doi:10.1128/mSphere.01044-20)
Supplement: TABLE S2 [file mSphere.01044-20-st002.docx]

| **Intake** | **ASD (*n* = 62)** | **TD (*n* = 20)** |
| --- | --- | --- |
| Total Energy (kcal) | 1527.65 ± 66.66 | 1484.31 ± 66.15 |
| Protein (g) | 51.5 ± 2.19 | 59.5 ± 3.38 |
| Fat (g) | 29.26 ± 4.06 | 31.02 ± 4.37 |
| Carbohydrate (g) | 264.59 ± 13.15 | 233.34 ± 15.91 |
| Fiber (g) | 9.01 ± 0.53 | 8.5 ± 0.81 |
| Vitamin A (ug) | 282.94 ± 50.46 | 333.85 ± 33.06 |
| Vitamin B1 (mg) | 0.86 ± 0.05 | 1.02 ± 0.08 |
| Vitamin B2 (mg)** | 0.54 ± 0.03 | 0.8 ± 0.07 |
| Vitamin C (mg) | 46.85 ± 7.00 | 41.21 ± 8.24 |
| Na (mg) | 455.72 ± 92.46 | 358.24 ± 39.46 |
| Ca (mg) | 395.42 ± 36.21 | 454.09 ± 53.05 |
| Mg (mg) | 212.7 ± 12.16 | 196.93 ± 10.72 |
| Fe (mg) | 13.16 ± 1 | 10.93 ± 1.46 |
| Zn (mg) | 7.4 ± 0.41 | 7.29 ± 0.69 |
