# Supplementary material for: Gut Bacteria Shared by Children and Their Mothers Associate with Developmental Level and Social Deficits in Autism Spectrum Disorder
Source: mSphere. 2020 Dec 2;5(6):e01044-20. doi: 10.1128/mSphere.01044-20 (PMC7716279; doi:10.1128/mSphere.01044-20)
Supplement: TABLE S3 [file mSphere.01044-20-st003.docx]

|  | **ASD (*n* = 70)** | **TD (*n* = 42)** |
| --- | --- | --- |
| GI symptoms/non** | 64/6 | 29/13 |
| Constipation/non** | 35/35 | 10/32 |
| Diarrhea/non | 11/59 | 4/38 |
| Abnormal stool consistency/non | 20/50 | 8/34 |
| Abnormal stool smell/non | 30/40 | 12/30 |
| Flatulence/non | 1/69 | 1/41 |
| Abdominal pain/non | 4/66 | 2/40 |
| Unexplained daytime irritability/non*** | 34/36 | 7/35 |
| Nighttime awakening/non | 30/40 | 14/28 |
| Abdominal tenderness (during examination)/non | 21/49 | 7/35 |
