# Supplementary figures and images for: Gut Bacteria Shared by Children and Their Mothers Associate with Developmental Level and Social Deficits in Autism Spectrum Disorder
Source: mSphere. 2020 Dec 2;5(6):e01044-20. doi: 10.1128/mSphere.01044-20 (PMC7716279; doi:10.1128/mSphere.01044-20)

**
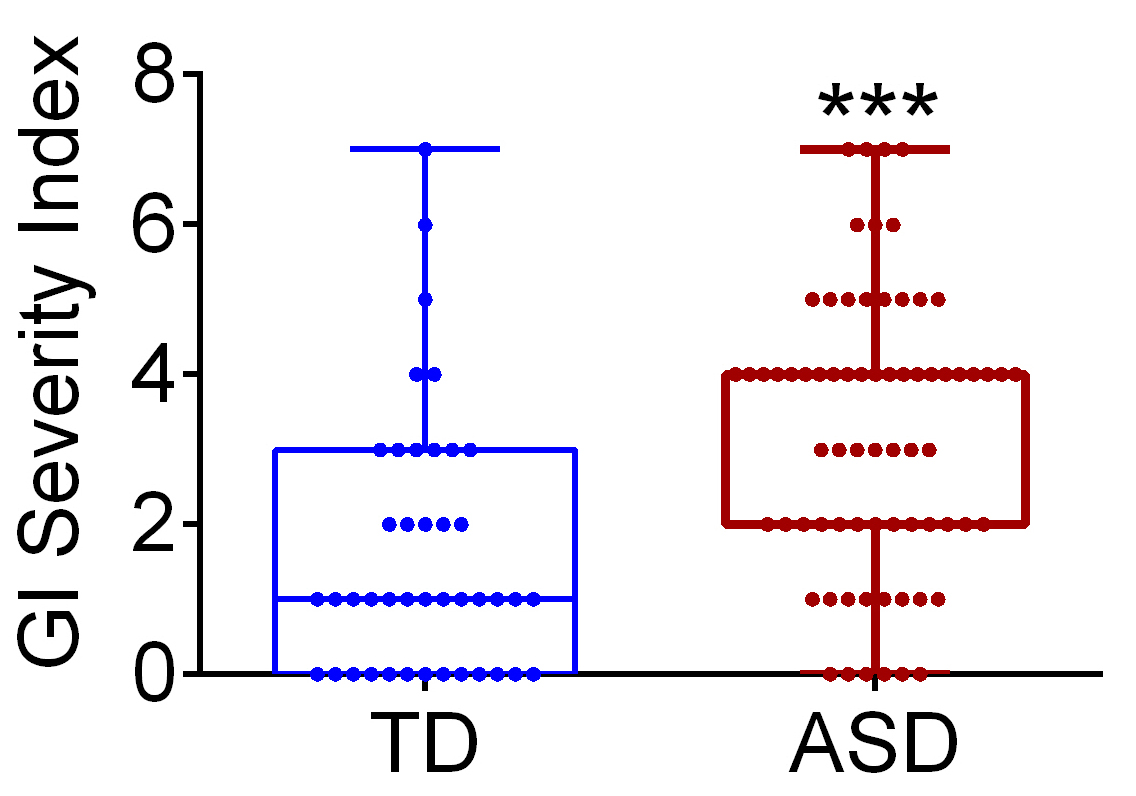
**

Supplement: FIG S1 [file mSphere.01044-20-sf001.docx]

**
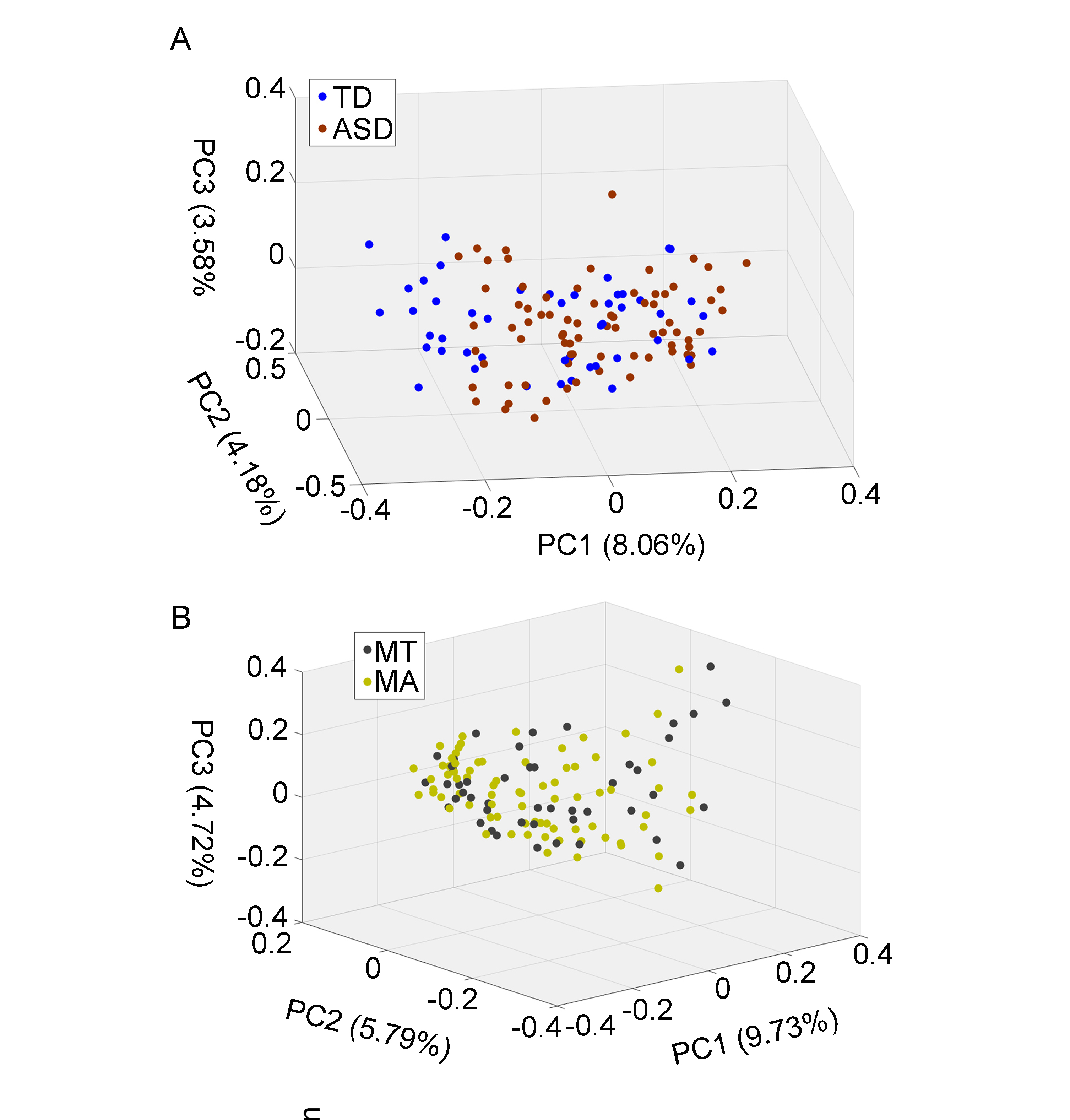
**

Supplement: FIG S2 [file mSphere.01044-20-sf002.docx]

**
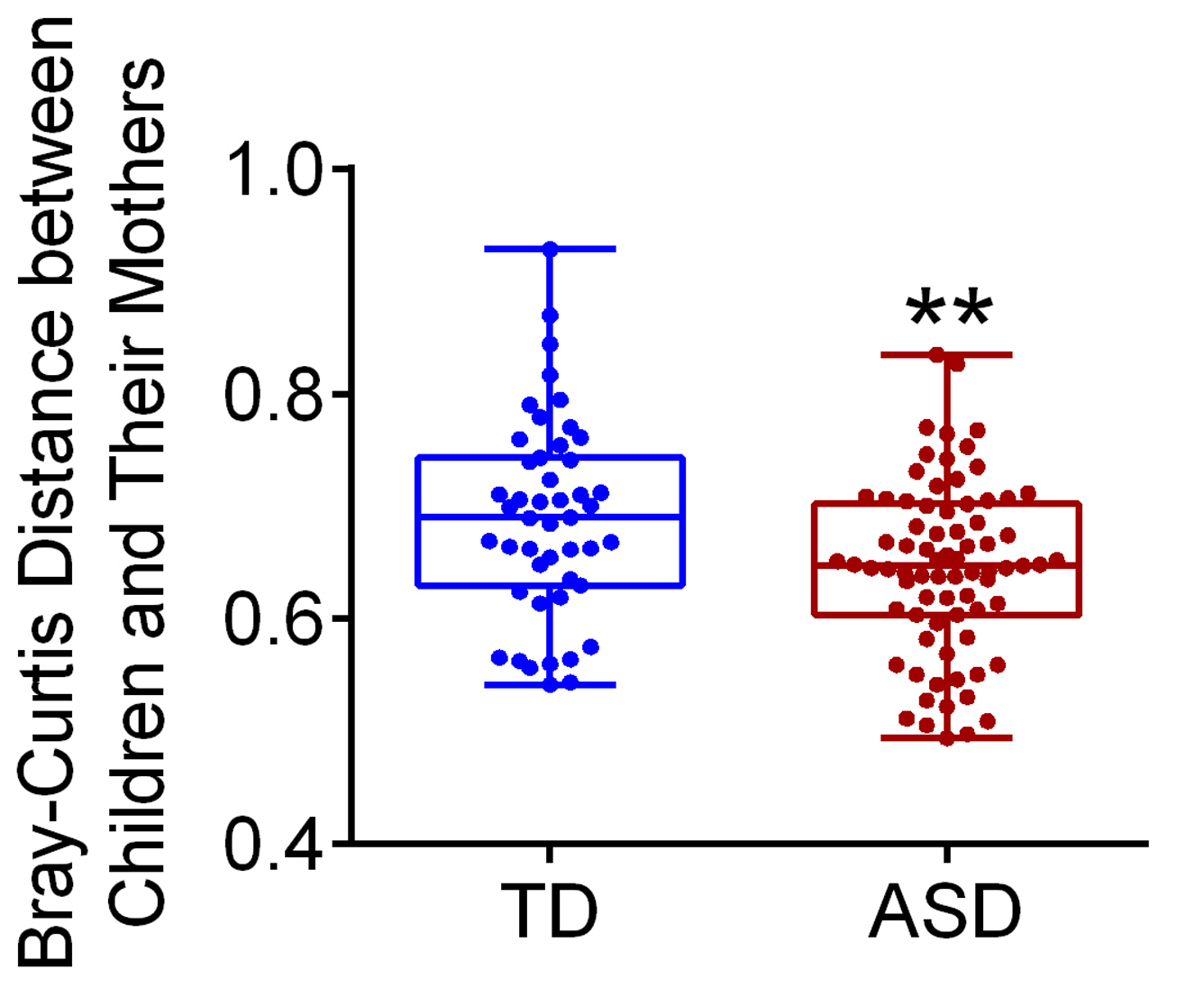
**

Supplement: FIG S3 [file mSphere.01044-20-sf003.docx]

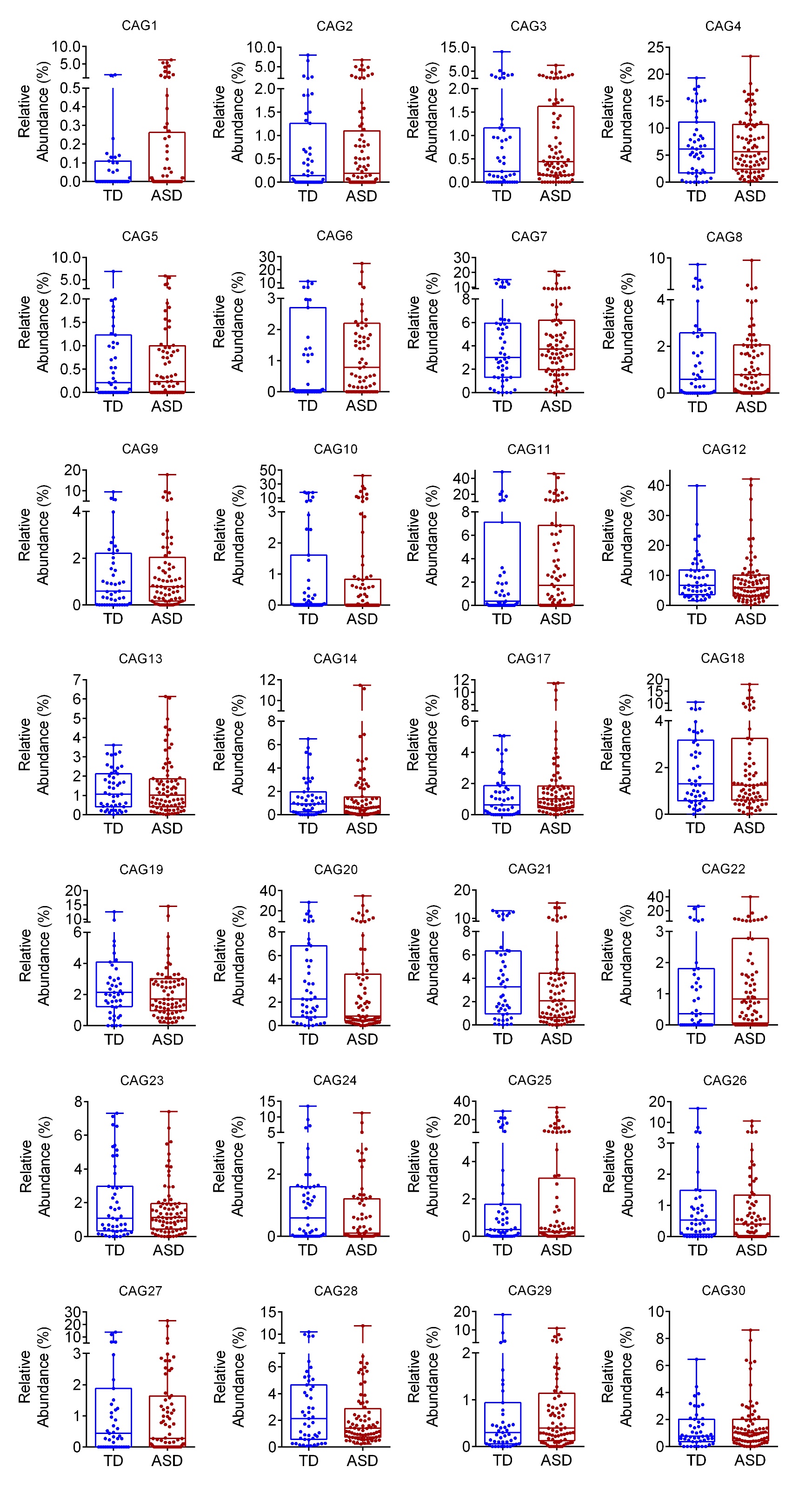

Supplement: FIG S4 [file mSphere.01044-20-sf004.docx]
