# Supplementary material for: Gut Bacteria Shared by Children and Their Mothers Associate with Developmental Level and Social Deficits in Autism Spectrum Disorder
Source: mSphere. 2020 Dec 2;5(6):e01044-20. doi: 10.1128/mSphere.01044-20 (PMC7716279; doi:10.1128/mSphere.01044-20)
Supplement: TABLE S4 [file mSphere.01044-20-st004.pdf]

ASVid	CAGid	Taxonomical assignments				
		Phylum	Class	Order	Family	Genus
ASV1339	CAG1	Actinobacteria	Actinobacteria	Bifidobacteriales	Bifidobacteriaceae	Bifidobacterium
ASV4789	CAG1	Firmicutes	Clostridia	Clostridiales	Ruminococcaceae	Butyricicoccus
ASV5288	CAG1	Firmicutes	Clostridia	Clostridiales	Christensenellaceae	Christensenellaceae R-7 group
ASV2602	CAG1	Firmicutes	Clostridia	Clostridiales	Family XIII	Family XIII UCG-001
ASV3349	CAG1	Firmicutes	Clostridia	Clostridiales	Ruminococcaceae	Ruminococcaceae UCG-002
ASV2157	CAG2	Firmicutes	Clostridia	Clostridiales	Lachnospiraceae	Eubacterium xylanophilum group
ASV4736	CAG2	Verrucomicrobia	Verrucomicrobiae	Verrucomicrobiales	Akkermansiaceae	Akkermansia
ASV2049	CAG2	Firmicutes	Clostridia	Clostridiales	Lachnospiraceae	Lachnospiraceae UCG-001
ASV4524	CAG2	Firmicutes	Clostridia	Clostridiales	Ruminococcaceae	Ruminococcaceae NK4A214 group
ASV4980	CAG2	Firmicutes	Clostridia	Clostridiales	Ruminococcaceae	Ruminococcus 1
ASV3194	CAG2	Firmicutes	Clostridia	Clostridiales	Ruminococcaceae	Ruminococcus 1
ASV990	CAG2	Firmicutes	Clostridia	Clostridiales	Lachnospiraceae	unclassified
ASV5425	CAG3	Bacteroidetes	Bacteroidia	Bacteroidales	Rikenellaceae	Alistipes
ASV4265	CAG3	Firmicutes	Clostridia	Clostridiales	Lachnospiraceae	Blautia
ASV3409	CAG3	Firmicutes	Clostridia	Clostridiales	Lachnospiraceae	CAG-56
ASV3765	CAG3	Firmicutes	Clostridia	Clostridiales	Christensenellaceae	Christensenellaceae R-7 group
ASV4144	CAG3	Firmicutes	Clostridia	Clostridiales	Lachnospiraceae	Coprococcus 3
ASV162	CAG3	Firmicutes	Clostridia	Clostridiales	Ruminococcaceae	Faecalibacterium
ASV4398	CAG3	Firmicutes	Clostridia	Clostridiales	Lachnospiraceae	Lachnospira
ASV3884	CAG3	Firmicutes	Clostridia	Clostridiales	Lachnospiraceae	Moryella
ASV1237	CAG3	Firmicutes	Clostridia	Clostridiales	Lachnospiraceae	uncultured
ASV2287	CAG4	Firmicutes	Clostridia	Clostridiales	Ruminococcaceae	Eubacterium coprostanoligenes group
ASV3740	CAG4	Firmicutes	Clostridia	Clostridiales	Lachnospiraceae	Blautia
ASV1017	CAG4	Firmicutes	Clostridia	Clostridiales	Lachnospiraceae	Blautia
ASV4172	CAG4	Firmicutes	Clostridia	Clostridiales	Christensenellaceae	Christensenellaceae R-7 group
ASV5092	CAG4	Firmicutes	Clostridia	Clostridiales	Lachnospiraceae	Dorea
ASV3999	CAG4	Firmicutes	Clostridia	Clostridiales	Ruminococcaceae	Faecalibacterium
ASV1478	CAG4	Firmicutes	Clostridia	Clostridiales	Ruminococcaceae	Faecalibacterium
ASV135	CAG4	Firmicutes	Clostridia	Clostridiales	Lachnospiraceae	Lachnospiraceae FCS020 group
ASV2223	CAG4	Firmicutes	Clostridia	Clostridiales	Lachnospiraceae	Lachnospiraceae ND3007 group
ASV1252	CAG4	Firmicutes	Clostridia	Clostridiales	Lachnospiraceae	Lachnospiraceae NK4A136 group
ASV531	CAG4	Firmicutes	Clostridia	Clostridiales	Lachnospiraceae	Lachnospiraceae NK4A136 group
ASV5368	CAG4	Firmicutes	Clostridia	Clostridiales	Lachnospiraceae	Lachnospiraceae UCG-004
ASV3701	CAG4	Firmicutes	Clostridia	Clostridiales	Ruminococcaceae	Ruminococcaceae UCG-003
ASV4247	CAG4	Firmicutes	Clostridia	Clostridiales	Ruminococcaceae	Subdoligranulum
ASV2416	CAG4	Firmicutes	Clostridia	Clostridiales	Lachnospiraceae	uncultured
ASV2422	CAG5	Firmicutes	Clostridia	Clostridiales	Lachnospiraceae	Lachnospiraceae NK4A136 group
ASV76	CAG5	Firmicutes	Clostridia	Clostridiales	Lachnospiraceae	Lachnospiraceae UCG-010
ASV1242	CAG5	Firmicutes	Clostridia	Clostridiales	Lachnospiraceae	Roseburia
ASV4132	CAG5	Firmicutes	Clostridia	Clostridiales	Ruminococcaceae	Ruminococcus 1
ASV1579	CAG5	Firmicutes	Clostridia	Clostridiales	Ruminococcaceae	Subdoligranulum
ASV563	CAG6	Firmicutes	Clostridia	Clostridiales	Lachnospiraceae	Eubacterium ruminantium group
ASV3814	CAG6	Firmicutes	Clostridia	Clostridiales	Lachnospiraceae	Agathobacter
ASV1588	CAG6	Firmicutes	Clostridia	Clostridiales	Ruminococcaceae	Ruminiclostridium 6
ASV3099	CAG6	Firmicutes	Clostridia	Clostridiales	Ruminococcaceae	Ruminococcaceae UCG-005
ASV205	CAG6	Firmicutes	Clostridia	Clostridiales	Ruminococcaceae	Ruminococcaceae UCG-014
ASV1707	CAG6	Firmicutes	Clostridia	Clostridiales	Lachnospiraceae	unclassified
ASV3014	CAG7	Firmicutes	Clostridia	Clostridiales	Ruminococcaceae	Eubacterium coprostanoligenes group
ASV276	CAG7	Proteobacteria	Deltaproteobacteria	Desulfovibrionales	Desulfovibrionaceae	Bilophila
ASV3605	CAG7	Firmicutes	Clostridia	Clostridiales	Christensenellaceae	Christensenellaceae R-7 group
ASV781	CAG7	Firmicutes	Clostridia	Clostridiales	Ruminococcaceae	Faecalibacterium
ASV4515	CAG7	Firmicutes	Clostridia	Clostridiales	Ruminococcaceae	Oscillibacter
ASV5369	CAG7	Firmicutes	Negativicutes	Selenomonadales	Acidaminococcaceae	Phascolarctobacterium
ASV3658	CAG7	Firmicutes	Clostridia	Clostridiales	Ruminococcaceae	Ruminiclostridium 5
ASV3851	CAG7	Firmicutes	Clostridia	Clostridiales	Ruminococcaceae	Ruminiclostridium 9
ASV1575	CAG7	Firmicutes	Clostridia	Clostridiales	Ruminococcaceae	Ruminococcaceae UCG-002
ASV2625	CAG7	Firmicutes	Clostridia	Clostridiales	Ruminococcaceae	Ruminococcaceae UCG-004
ASV709	CAG7	Firmicutes	Clostridia	Clostridiales	Lachnospiraceae	uncultured
ASV1238	CAG7	Firmicutes	Clostridia	Clostridiales	Lachnospiraceae	uncultured
ASV284	CAG8	Bacteroidetes	Bacteroidia	Bacteroidales	Rikenellaceae	Alistipes
ASV4563	CAG8	Bacteroidetes	Bacteroidia	Bacteroidales	Rikenellaceae	Alistipes
ASV1772	CAG8	Bacteroidetes	Bacteroidia	Bacteroidales	Rikenellaceae	Alistipes
ASV4080	CAG8	Firmicutes	Clostridia	Clostridiales	Ruminococcaceae	Ruminococcaceae UCG-002
ASV4791	CAG8	Firmicutes	Clostridia	Clostridiales	Ruminococcaceae	Ruminococcaceae UCG-003
ASV1355	CAG8	Firmicutes	Clostridia	Clostridiales	Ruminococcaceae	Ruminococcaceae UCG-005
ASV3893	CAG8	Firmicutes	Clostridia	Clostridiales	Ruminococcaceae	Subdoligranulum
ASV5192	CAG8	Firmicutes	Clostridia	Clostridiales	Ruminococcaceae	uncultured
ASV5300	CAG9	Firmicutes	Clostridia	Clostridiales	Ruminococcaceae	Eubacterium coprostanoligenes group
ASV1138	CAG9	Firmicutes	Clostridia	Clostridiales	Ruminococcaceae	Eubacterium coprostanoligenes group
ASV764	CAG9	Bacteroidetes	Bacteroidia	Bacteroidales	Rikenellaceae	Alistipes
ASV4313	CAG9	Bacteroidetes	Bacteroidia	Bacteroidales	Rikenellaceae	Alistipes
ASV4441	CAG9	Bacteroidetes	Bacteroidia	Bacteroidales	Rikenellaceae	Alistipes
ASV4570	CAG9	Firmicutes	Clostridia	Clostridiales	Christensenellaceae	Christensenellaceae R-7 group
ASV3712	CAG9	Firmicutes	Clostridia	Clostridiales	Lachnospiraceae	Dorea
ASV3527	CAG9	Bacteroidetes	Bacteroidia	Bacteroidales	Marinifilaceae	Odoribacter
ASV1240	CAG9	Bacteroidetes	Bacteroidia	Bacteroidales	Marinifilaceae	Odoribacter
ASV1255	CAG9	Firmicutes	Clostridia	Clostridiales	Ruminococcaceae	Ruminiclostridium 5
ASV3995	CAG9	Firmicutes	Clostridia	Clostridiales	Ruminococcaceae	Ruminiclostridium 9
ASV692	CAG10	Bacteroidetes	Bacteroidia	Bacteroidales	Bacteroidaceae	Bacteroides
ASV404	CAG10	Bacteroidetes	Bacteroidia	Bacteroidales	Bacteroidaceae	Bacteroides
ASV3001	CAG10	Bacteroidetes	Bacteroidia	Bacteroidales	Tannerellaceae	Parabacteroides
ASV617	CAG10	Bacteroidetes	Bacteroidia	Bacteroidales	Tannerellaceae	Parabacteroides
ASV744	CAG10	Bacteroidetes	Bacteroidia	Bacteroidales	Tannerellaceae	Parabacteroides
ASV117	CAG10	Bacteroidetes	Bacteroidia	Bacteroidales	Tannerellaceae	Parabacteroides
ASV1470	CAG11	Bacteroidetes	Bacteroidia	Bacteroidales	Rikenellaceae	Alistipes
ASV1874	CAG11	Bacteroidetes	Bacteroidia	Bacteroidales	Bacteroidaceae	Bacteroides
ASV3904	CAG11	Bacteroidetes	Bacteroidia	Bacteroidales	Bacteroidaceae	Bacteroides
ASV5175	CAG11	Bacteroidetes	Bacteroidia	Bacteroidales	Bacteroidaceae	Bacteroides
ASV5334	CAG11	Firmicutes	Negativicutes	Selenomonadales	Veillonellaceae	Megamonas
ASV4096	CAG11	Firmicutes	Negativicutes	Selenomonadales	Veillonellaceae	Megamonas
ASV2208	CAG11	Firmicutes	Negativicutes	Selenomonadales	Veillonellaceae	Megamonas
ASV1666	CAG11	Bacteroidetes	Bacteroidia	Bacteroidales	Tannerellaceae	Parabacteroides
ASV2636	CAG11	Bacteroidetes	Bacteroidia	Bacteroidales	Prevotellaceae	Prevotella 9
ASV2311	CAG11	Bacteroidetes	Bacteroidia	Bacteroidales	Prevotellaceae	Prevotella 9
ASV3031	CAG11	Bacteroidetes	Bacteroidia	Bacteroidales	Prevotellaceae	Prevotella 9
ASV1672	CAG11	Bacteroidetes	Bacteroidia	Bacteroidales	Prevotellaceae	Prevotella 9
ASV2646	CAG12	Firmicutes	Erysipelotrichia	Erysipelotrichales	Erysipelotrichaceae	Clostridium innocuum group

ASV4252	CAG12	Firmicutes	Clostridia	Clostridiales	Ruminococcaceae	Faecalibacterium
ASV3328	CAG12	Firmicutes	Clostridia	Clostridiales	Ruminococcaceae	Flavonifractor
ASV2613	CAG12	Firmicutes	Clostridia	Clostridiales	Lachnospiraceae	Hungatella
ASV937	CAG12	Firmicutes	Clostridia	Clostridiales	Lachnospiraceae	Lachnoclostridium
ASV2704	CAG12	Firmicutes	Clostridia	Clostridiales	Lachnospiraceae	Lachnoclostridium
ASV731	CAG12	Firmicutes	Clostridia	Clostridiales	Lachnospiraceae	Tyzzerella
ASV942	CAG12	Firmicutes	Clostridia	Clostridiales	Lachnospiraceae	unclassified
ASV3991	CAG13	Firmicutes	Clostridia	Clostridiales	Lachnospiraceae	Ruminococcus torques group
ASV330	CAG13	Firmicutes	Clostridia	Clostridiales	Ruminococcaceae	Anaerotruncus
ASV1261	CAG13	Firmicutes	Clostridia	Clostridiales	Ruminococcaceae	DTU089
ASV161	CAG13	Actinobacteria	Coriobacteriia	Coriobacteriales	Eggerthellaceae	Eggerthella
ASV1989	CAG13	Firmicutes	Clostridia	Clostridiales	Lachnospiraceae	Eisenbergiella
ASV4859	CAG13	Firmicutes	Clostridia	Clostridiales	Lachnospiraceae	Lachnoclostridium
ASV4522	CAG13	Firmicutes	Clostridia	Clostridiales	Lachnospiraceae	Lachnoclostridium
ASV228	CAG13	Firmicutes	Clostridia	Clostridiales	Ruminococcaceae	Oscillibacter
ASV5440	CAG13	Firmicutes	Clostridia	Clostridiales	Ruminococcaceae	Ruminiclostridium 9
ASV2314	CAG13	Firmicutes	Clostridia	Clostridiales	Lachnospiraceae	Sellimonas
ASV2810	CAG13	Firmicutes	Clostridia	Clostridiales	Lachnospiraceae	Tyzzerella
ASV2337	CAG13	Firmicutes	Clostridia	Clostridiales	Lachnospiraceae	Tyzzerella 4
ASV1678	CAG13	Firmicutes	Clostridia	Clostridiales	Ruminococcaceae	UBA1819
ASV3447	CAG14	Firmicutes	Clostridia	Clostridiales	Lachnospiraceae	Blautia
ASV1155	CAG14	Firmicutes	Negativicutes	Selenomonadales	Veillonellaceae	Dialister
ASV5443	CAG14	Proteobacteria	Gammaproteobacteria	Enterobacteriales	Enterobacteriaceae	Escherichia-Shigella
ASV4828	CAG14	Proteobacteria	Gammaproteobacteria	Enterobacteriales	Enterobacteriaceae	Escherichia-Shigella
ASV4562	CAG14	Firmicutes	Negativicutes	Selenomonadales	Veillonellaceae	Veillonella
ASV406	CAG15	Firmicutes	Clostridia	Clostridiales	Lachnospiraceae	Ruminococcus gnavus group
ASV2839	CAG15	Firmicutes	Clostridia	Clostridiales	Lachnospiraceae	Blautia
ASV3051	CAG15	Firmicutes	Clostridia	Clostridiales	Lachnospiraceae	Blautia
ASV4291	CAG15	Actinobacteria	Coriobacteriia	Coriobacteriales	Coriobacteriaceae	Collinsella
ASV3792	CAG15	Actinobacteria	Coriobacteriia	Coriobacteriales	Eggerthellaceae	Eggerthella
ASV493	CAG15	Firmicutes	Erysipelotrichia	Erysipelotrichales	Erysipelotrichaceae	Erysipelatoclostridium
ASV4196	CAG15	Fusobacteria	Fusobacteriia	Fusobacteriales	Fusobacteriaceae	Fusobacterium
ASV790	CAG15	Firmicutes	Clostridia	Clostridiales	Lachnospiraceae	Lachnoclostridium
ASV1765	CAG15	Firmicutes	Clostridia	Clostridiales	Lachnospiraceae	Tyzzerella 4
ASV497	CAG15	Firmicutes	Clostridia	Clostridiales	Lachnospiraceae	unclassified
ASV4568	CAG15	Firmicutes	Clostridia	Clostridiales	Lachnospiraceae	unclassified
ASV3419	CAG15	Firmicutes	Clostridia	Clostridiales	Lachnospiraceae	unclassified
ASV2875	CAG16	Bacteroidetes	Bacteroidia	Bacteroidales	Bacteroidaceae	Bacteroides
ASV3945	CAG16	Bacteroidetes	Bacteroidia	Bacteroidales	Bacteroidaceae	Bacteroides
ASV3889	CAG16	Bacteroidetes	Bacteroidia	Bacteroidales	Bacteroidaceae	Bacteroides
ASV4348	CAG16	Firmicutes	Clostridia	Clostridiales	Lachnospiraceae	Lachnoclostridium
ASV3543	CAG16	Firmicutes	Bacilli	Lactobacillales	Streptococcaceae	Streptococcus
ASV2575	CAG16	Firmicutes	Clostridia	Clostridiales	Lachnospiraceae	unclassified
ASV1786	CAG17	Firmicutes	Clostridia	Clostridiales	Lachnospiraceae	Eubacterium hallii group
ASV1085	CAG17	Firmicutes	Clostridia	Clostridiales	Lachnospiraceae	Eubacterium ventriosum group
ASV5280	CAG17	Firmicutes	Clostridia	Clostridiales	Lachnospiraceae	Agathobacter
ASV2090	CAG17	Bacteroidetes	Bacteroidia	Bacteroidales	Bacteroidaceae	Bacteroides
ASV4017	CAG17	Firmicutes	Clostridia	Clostridiales	Lachnospiraceae	Coprococcus 1
ASV4218	CAG17	Firmicutes	Erysipelotrichia	Erysipelotrichales	Erysipelotrichaceae	Erysipelotrichaceae UCG-003
ASV728	CAG17	Firmicutes	Clostridia	Clostridiales	Lachnospiraceae	Lachnoclostridium
ASV547	CAG17	Firmicutes	Clostridia	Clostridiales	Lachnospiraceae	Lachnospiraceae UCG-001
ASV4678	CAG17	Firmicutes	Negativicutes	Selenomonadales	Veillonellaceae	Megasphaera
ASV838	CAG17	Firmicutes	Clostridia	Clostridiales	Lachnospiraceae	Roseburia
ASV4833	CAG17	Firmicutes	Clostridia	Clostridiales	Lachnospiraceae	Tyzzerella 3
ASV4746	CAG18	Verrucomicrobia	Verrucomicrobiae	Verrucomicrobiales	Akkermansiaceae	Akkermansia
ASV3116	CAG18	Bacteroidetes	Bacteroidia	Bacteroidales	Bacteroidaceae	Bacteroides
ASV4148	CAG18	Actinobacteria	Actinobacteria	Bifidobacteriales	Bifidobacteriaceae	Bifidobacterium
ASV1203	CAG18	Actinobacteria	Actinobacteria	Bifidobacteriales	Bifidobacteriaceae	Bifidobacterium
ASV1306	CAG18	Actinobacteria	Actinobacteria	Bifidobacteriales	Bifidobacteriaceae	Bifidobacterium
ASV5266	CAG18	Actinobacteria	Actinobacteria	Bifidobacteriales	Bifidobacteriaceae	Bifidobacterium
ASV485	CAG18	Actinobacteria	Coriobacteriia	Coriobacteriales	Coriobacteriaceae	Collinsella
ASV1143	CAG19	Firmicutes	Clostridia	Clostridiales	Lachnospiraceae	Eubacterium hallii group
ASV1904	CAG19	Firmicutes	Clostridia	Clostridiales	Lachnospiraceae	Eubacterium ventriosum group
ASV3137	CAG19	Firmicutes	Clostridia	Clostridiales	Lachnospiraceae	Ruminococcus torques group
ASV2018	CAG19	Firmicutes	Clostridia	Clostridiales	Lachnospiraceae	Agathobacter
ASV1070	CAG19	Firmicutes	Clostridia	Clostridiales	Lachnospiraceae	Anaerostipes
ASV3372	CAG19	Firmicutes	Clostridia	Clostridiales	Lachnospiraceae	Fusicatenibacter
ASV5269	CAG19	Firmicutes	Clostridia	Clostridiales	Lachnospiraceae	Lachnospira
ASV3246	CAG19	Bacteroidetes	Bacteroidia	Bacteroidales	Tannerellaceae	Parabacteroides
ASV3620	CAG19	Proteobacteria	Gammaproteobacteria	Betaproteobacteriales	Burkholderiaceae	Parasutterella
ASV981	CAG19	Proteobacteria	Gammaproteobacteria	Betaproteobacteriales	Burkholderiaceae	Parasutterella
ASV2123	CAG20	Bacteroidetes	Bacteroidia	Bacteroidales	Bacteroidaceae	Bacteroides
ASV1759	CAG20	Firmicutes	Clostridia	Clostridiales	Ruminococcaceae	Flavonifractor
ASV411	CAG20	Firmicutes	Erysipelotrichia	Erysipelotrichales	Erysipelotrichaceae	Holdemania
ASV4464	CAG20	Firmicutes	Clostridia	Clostridiales	Ruminococcaceae	Intestinimonas
ASV4143	CAG20	Proteobacteria	Gammaproteobacteria	Enterobacteriales	Enterobacteriaceae	Klebsiella
ASV828	CAG20	Firmicutes	Clostridia	Clostridiales	Lachnospiraceae	Lachnoclostridium
ASV584	CAG20	Firmicutes	Clostridia	Clostridiales	Lachnospiraceae	Lachnospiraceae UCG-010
ASV927	CAG20	Firmicutes	Clostridia	Clostridiales	Ruminococcaceae	Ruminiclostridium 9
ASV831	CAG20	Firmicutes	Clostridia	Clostridiales	Lachnospiraceae	unclassified
ASV3988	CAG20	Firmicutes	Clostridia	Clostridiales	Lachnospiraceae	uncultured
ASV660	CAG20	Firmicutes	Clostridia	Clostridiales	Lachnospiraceae	uncultured
ASV714	CAG21	Firmicutes	Clostridia	Clostridiales	Lachnospiraceae	Eubacterium eligens group
ASV260	CAG21	Firmicutes	Clostridia	Clostridiales	Lachnospiraceae	Anaerostipes
ASV1545	CAG21	Bacteroidetes	Bacteroidia	Bacteroidales	Bacteroidaceae	Bacteroides
ASV853	CAG21	Bacteroidetes	Bacteroidia	Bacteroidales	Bacteroidaceae	Bacteroides
ASV2430	CAG21	Bacteroidetes	Bacteroidia	Bacteroidales	Bacteroidaceae	Bacteroides
ASV1105	CAG21	Firmicutes	Clostridia	Clostridiales	Ruminococcaceae	Caproiciproducens
ASV3067	CAG21	Firmicutes	Clostridia	Clostridiales	Lachnospiraceae	Eisenbergiella
ASV5356	CAG21	Firmicutes	Clostridia	Clostridiales	Ruminococcaceae	Faecalibacterium
ASV874	CAG21	Synergistetes	Synergistia	Synergistales	Synergistaceae	Pyramidobacter
ASV3804	CAG21	Firmicutes	Clostridia	Clostridiales	Ruminococcaceae	Ruminiclostridium 5
ASV2999	CAG21	Firmicutes	Clostridia	Clostridiales	Ruminococcaceae	Ruminococcus 2
ASV5248	CAG22	Firmicutes	Clostridia	Clostridiales	Lachnospiraceae	Eubacterium eligens group
ASV514	CAG22	Firmicutes	Clostridia	Clostridiales	Lachnospiraceae	Eubacterium eligens group
ASV4525	CAG22	Bacteroidetes	Bacteroidia	Bacteroidales	Bacteroidaceae	Bacteroides
ASV3963	CAG22	Firmicutes	Clostridia	Clostridiales	Ruminococcaceae	Faecalibacterium
ASV4836	CAG22	Firmicutes	Clostridia	Clostridiales	Ruminococcaceae	Faecalibacterium
ASV1689	CAG22	Firmicutes	Clostridia	Clostridiales	Ruminococcaceae	uncultured
ASV1846	CAG23	Firmicutes	Clostridia	Clostridiales	Lachnospiraceae	Eubacterium ventriosum group

ASV1246	CAG23	Firmicutes	Clostridia	Clostridiales	Lachnospiraceae	Ambiguous_taxa
ASV4830	CAG23	Firmicutes	Clostridia	Clostridiales	Ruminococcaceae	Butyricicoccus
ASV4908	CAG23	Firmicutes	Clostridia	Clostridiales	Lachnospiraceae	Lachnospira
ASV4883	CAG23	Bacteroidetes	Bacteroidia	Bacteroidales	Tannerellaceae	Parabacteroides
ASV1500	CAG23	Firmicutes	Clostridia	Clostridiales	Ruminococcaceae	Ruminococcaceae UCG-013
ASV4366	CAG23	Firmicutes	Clostridia	Clostridiales	Lachnospiraceae	Tyzzerella
ASV1782	CAG24	Firmicutes	Clostridia	Clostridiales	Lachnospiraceae	Ruminococcus torques group
ASV3240	CAG24	Bacteroidetes	Bacteroidia	Bacteroidales	Bacteroidaceae	Bacteroides
ASV1616	CAG24	Bacteroidetes	Bacteroidia	Bacteroidales	Bacteroidaceae	Bacteroides
ASV1649	CAG24	Bacteroidetes	Bacteroidia	Bacteroidales	Tannerellaceae	Parabacteroides
ASV1343	CAG24	Firmicutes	Clostridia	Clostridiales	Ruminococcaceae	Phocea
ASV4245	CAG24	Firmicutes	Clostridia	Clostridiales	Ruminococcaceae	Ruminococcus 1
ASV1189	CAG24	Proteobacteria	Gammaproteobacteria	Betaproteobacteriales	Burkholderiaceae	Sutterella
ASV1015	CAG25	Firmicutes	Clostridia	Clostridiales	Lachnospiraceae	Anaerostipes
ASV1602	CAG25	Bacteroidetes	Bacteroidia	Bacteroidales	Bacteroidaceae	Bacteroides
ASV3813	CAG25	Bacteroidetes	Bacteroidia	Bacteroidales	Bacteroidaceae	Bacteroides
ASV4961	CAG25	Bacteroidetes	Bacteroidia	Bacteroidales	Bacteroidaceae	Bacteroides
ASV5209	CAG25	Bacteroidetes	Bacteroidia	Bacteroidales	Bacteroidaceae	Bacteroides
ASV3736	CAG25	Firmicutes	Clostridia	Clostridiales	Ruminococcaceae	Flavonifractor
ASV708	CAG25	Firmicutes	Erysipelotrichia	Erysipelotrichales	Erysipelotrichaceae	Holdemania
ASV5148	CAG25	Firmicutes	Clostridia	Clostridiales	Ruminococcaceae	Intestinimonas
ASV3592	CAG26	Firmicutes	Clostridia	Clostridiales	Lachnospiraceae	Agathobacter
ASV953	CAG26	Bacteroidetes	Bacteroidia	Bacteroidales	Bacteroidaceae	Bacteroides
ASV163	CAG26	Bacteroidetes	Bacteroidia	Bacteroidales	Bacteroidaceae	Bacteroides
ASV4387	CAG26	Bacteroidetes	Bacteroidia	Bacteroidales	Bacteroidaceae	Bacteroides
ASV1909	CAG26	Bacteroidetes	Bacteroidia	Bacteroidales	Bacteroidaceae	Bacteroides
ASV3898	CAG26	Firmicutes	Clostridia	Clostridiales	Clostridiaceae 1	Clostridium sensu stricto 1
ASV5304	CAG26	Firmicutes	Clostridia	Clostridiales	Ruminococcaceae	Ruminococcus 2
ASV577	CAG26	Firmicutes	Clostridia	Clostridiales	Lachnospiraceae	unclassified
ASV475	CAG27	Bacteroidetes	Bacteroidia	Bacteroidales	Bacteroidaceae	Bacteroides
ASV5119	CAG27	Bacteroidetes	Bacteroidia	Bacteroidales	Bacteroidaceae	Bacteroides
ASV3777	CAG27	Bacteroidetes	Bacteroidia	Bacteroidales	Bacteroidaceae	Bacteroides
ASV4802	CAG27	Bacteroidetes	Bacteroidia	Bacteroidales	Bacteroidaceae	Bacteroides
ASV1768	CAG27	Bacteroidetes	Bacteroidia	Bacteroidales	Bacteroidaceae	Bacteroides
ASV54	CAG27	Bacteroidetes	Bacteroidia	Bacteroidales	Bacteroidaceae	Bacteroides
ASV4488	CAG27	Bacteroidetes	Bacteroidia	Bacteroidales	Bacteroidaceae	Bacteroides
ASV2130	CAG28	Firmicutes	Clostridia	Clostridiales	Ruminococcaceae	Butyricicoccus
ASV5202	CAG28	Firmicutes	Clostridia	Clostridiales	Clostridiaceae 1	Clostridium sensu stricto 1
ASV3864	CAG28	Firmicutes	Clostridia	Clostridiales	Clostridiaceae 1	Clostridium sensu stricto 1
ASV2899	CAG28	Firmicutes	Clostridia	Clostridiales	Clostridiaceae 1	Clostridium sensu stricto 1
ASV543	CAG28	Proteobacteria	Gammaproteobacteria	Pasteurellales	Pasteurellaceae	Haemophilus
ASV3390	CAG28	Firmicutes	Clostridia	Clostridiales	Peptostreptococcaceae	Intestinibacter
ASV1016	CAG28	Firmicutes	Clostridia	Clostridiales	Lachnospiraceae	Lachnospiraceae UCG-004
ASV4733	CAG28	Firmicutes	Clostridia	Clostridiales	Peptostreptococcaceae	Romboutsia
ASV2671	CAG28	Firmicutes	Clostridia	Clostridiales	Lachnospiraceae	Roseburia
ASV2373	CAG28	Firmicutes	Bacilli	Lactobacillales	Streptococcaceae	Streptococcus
ASV1717	CAG28	Firmicutes	Erysipelotrichia	Erysipelotrichales	Erysipelotrichaceae	Turicibacter
ASV3556	CAG29	Firmicutes	Bacilli	Lactobacillales	Carnobacteriaceae	Granulicatella
ASV1664	CAG29	Firmicutes	Clostridia	Clostridiales	Lachnospiraceae	Lachnoclostridium
ASV4622	CAG29	Firmicutes	Clostridia	Clostridiales	Lachnospiraceae	Roseburia
ASV1378	CAG29	Firmicutes	Bacilli	Lactobacillales	Streptococcaceae	Streptococcus
ASV4545	CAG29	Firmicutes	Negativicutes	Selenomonadales	Veillonellaceae	Veillonella
ASV808	CAG29	Firmicutes	Negativicutes	Selenomonadales	Veillonellaceae	Veillonella
ASV1850	CAG29	Firmicutes	Negativicutes	Selenomonadales	Veillonellaceae	Veillonella
ASV2005	CAG29	Firmicutes	Negativicutes	Selenomonadales	Veillonellaceae	Veillonella
ASV537	CAG30	Firmicutes	Clostridia	Clostridiales	Lachnospiraceae	Eubacterium eligens group
ASV2507	CAG30	Bacteroidetes	Bacteroidia	Bacteroidales	Bacteroidaceae	Bacteroides
ASV45	CAG30	Bacteroidetes	Bacteroidia	Bacteroidales	Bacteroidaceae	Bacteroides
ASV1359	CAG30	Firmicutes	Clostridia	Clostridiales	Lachnospiraceae	Blautia
ASV526	CAG30	Firmicutes	Clostridia	Clostridiales	Ruminococcaceae	Butyricicoccus
ASV4195	CAG30	Firmicutes	Clostridia	Clostridiales	Lachnospiraceae	Lachnospiraceae UCG-004
ASV3619	CAG30	Bacteroidetes	Bacteroidia	Bacteroidales	Tannerellaceae	Parabacteroides
ASV337	CAG30	Proteobacteria	Gammaproteobacteria	Betaproteobacteriales	Burkholderiaceae	Parasutterella
ASV3232	CAG30	Proteobacteria	Gammaproteobacteria	Betaproteobacteriales	Burkholderiaceae	Parasutterella
